# Triglyceride-glucose index as an independent predictor of mortality in patients with chronic respiratory diseases

**DOI:** 10.3389/fphar.2025.1474265

**Published:** 2025-05-12

**Authors:** Hongyu Lu, Jibo Li, Xinlong Liu, Pan Jiang, Yongwen Feng, Changshan Wang, Feng Xu

**Affiliations:** ^1^ Department of Intensive Care Unit, Shenzhen Guangming District People’s Hospital, Shenzhen, Guangdong, China; ^2^ Department of Stomatology, Shenzhen Guangming District People’s Hospital, Shenzhen, Guangdong, China; ^3^ Institutes for Translational Medicine, Shenzhen Guangming District People’s Hospital, Shenzhen, Guangdong, China

**Keywords:** triglyceride-glucose index, mortality, chronic respiratory diseases, NHANES, risk prediction

## Abstract

**Objective:**

The consequences of chronic pulmonary illness are known to exacerbate in individuals with metabolic syndrome and insulin resistance. However, the relationship between triglyceride-glucose (TyG) index, a reliable alternative biomarker of metabolic dysfunction, and chronic respiratory diseases (CRDs) are inconclusive.

**Research design and methods:**

Our research involved a total of 7,819 adult individuals diagnosed with CRDs who participated in the National Health and Nutrition Examination Survey (NHANES) from 2001 to 2018. To assess the correlation between the TyG index and survival rates, we employed multivariable weighted Cox regression analysis, smoothing curve fitting, survival curve analysis and subgroup analysis to investigate the relationship.

**Results:**

Higher TyG index among CRDs shown a substantial positive correlation with all-cause mortality after controlling for relevant confounders. The restricted cubic spline analysis showed a nonlinear relationship between the TyG score and all-cause mortality in CRDs. Patients with higher TyG indexes had a greater risk of all-cause mortality according to Kaplan-Meier survival curves.

**Conclusion:**

The clinical relevance of the TyG index in predicting the life expectancy of individuals with CRDs is highlighted by our research. The TyG index can serve as a substitute biomarker for monitoring the wellbeing of the individuals with CRDs.

## Introduction

Chronic Respiratory Diseases (CRDs) involve of several lung and airway diseases, primarily including chronic bronchitis, emphysema and asthma (Global burden of chronic respiratory diseases and risk factors, 2023). They have a significant impact on global morbidity and mortality (The, 2018). As shown in the Global Burden of Disease study, nearly nine million people worldwide die from CRDs every year, accounting for 7% of total global deaths (Prevalence and attributable health burden of chronic respiratory diseases, 2020). Additionally, in 2019, the number of people diagnosed with CRDs rose rapidly, with an estimated 454.6 million cases (Global burden of chronic respiratory diseases and risk factors, 2023; Global, 2017). This has significantly adverse effects on quality of life and mortality risks, leading to a major burden on global healthcare, society, and the economy (GBD, 2019 Diseases and Injuries Collaborators, 2020; Chen et al., 2023).

Previous research has demonstrated that Triglyceride-Glucose (TyG) index and fasting blood glucose (FBG) can function as proxy biochemical signs to evaluate insulin resistance. According to recent studies, those with insulin resistance who also have asthma may have poorer response to treatment, faster lung function loss, and less lung function (Gläser et al., 2015). Furthermore, in healthy individuals, insulin resistance has been found to be a substantial risk factor for decreased pulmonary function (Ehrlich et al., 2010). Reduced lung function in healthy individuals has been associated with the presence of insulin resistance, which serves as a significant risk indicator. By investigating whether the TyG index independently influences CRDs patients, this study aims to identify individuals at high risk of all-cause mortality and potentially intervene early. To accomplish this goal, data from a nationally representative sample of individuals with CRDs were extracted from the US National Health and Nutrition Examination Survey (NHANES).

## Research design and methods

### Study population

**FIGURE 1:**
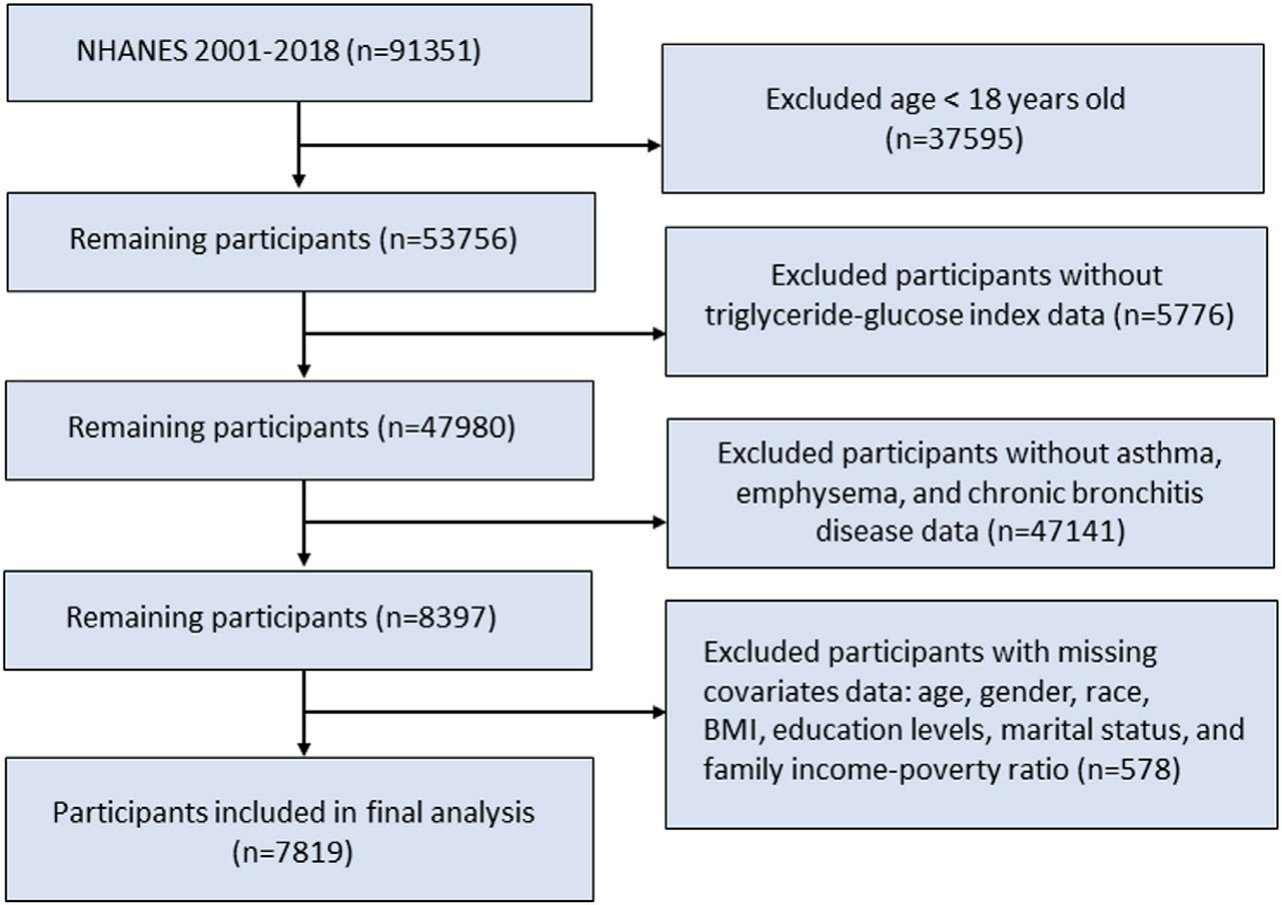
Flow chart of study population selection.

The NHANES offered a comprehensive analysis of the nutritional and health conditions within the US non-institutionalized civilian population. Our study utilized data from the continuous NHANES survey from eleven survey cycles, conducted from 2001 to 2018 with a total of 91,351 participants. A total of 53,756 eligible participants aged 18 years and older were included in the study population. We next excluded patients without TyG index or CRDs disease data. Additionally, participants without records of age, sex, race, marital status, education level, body mass index, and family income-poverty ratio were excluded. In the end, a total of 7,819 individuals were enrolled in this research (Figure 1).

### Evaluation of mortality

The data on mortality from all causes was obtained by connecting with the National Death Index until 26 April 2022.

### Statistical analysis

The TyG index was determined by taking the natural logarithm of the product of glucose and triglyceride levels, both measured in mg/dL. TyG index was calculated using the formula Ln [fasting triglycerides (mg/dL) × fasting glucose (mg/dL)]/2 (Simental-Mendía and Guerrero-Romero, 2020). Participants were equally classified into three groups based on the values of TyG index. The Cox proportional hazards regression model was utilized to compute and present the adjusted hazard ratios (HRs) along with their corresponding 95% confidence intervals (95% CIs). The current observational study utilized three models following the guidelines of Strengthening the Reporting of Observational Studies in Epidemiology (STROBE). An initial model was adjusted for none (model 1). Model two was adjusted for age, sex, and race. Model 3 was adjusted for all variables in model two plus other risk factors for marital status, education level, body mass index, and family income-poverty ratio levels. Kaplan–Meier survival curves for all-cause mortality stratified by the values of TyG index was generated. We used a penalized spline method for smooth curve fitting in order to examine any possible non-linear association between TyG index and all-cause mortality. A P value of less than 0.05 is considered statistically significant. All statistical analyses were performed with R version 4.2.2 and Free Statistics software version V2.1Beta.

## Results

### Subject characteristics

The baseline characteristics of the analysis cohort were shown in Table 1 according to the changes of TyG index among all groups. This analysis included 7,819 patients. Compared to the lowest TyG index group, patients in the highest TyG index tertile had a higher average age and BMI, were mostly unmarried, had a lower level of education and family income-poverty ratio.

**TABLE 1:** Demographic and clinical characteristics according to triglyceride-glucose index level.

Variables	TyG index				P-value
	Total	Q1	Q2	Q3	
Number of patients					
TyG	4.9 ± 0.2	4.7 ± 0.1	4.9 ± 0.0	5.1 ± 0.2	<0.001
Age (years)	49.9 ± 18.4	44.5 ± 18.8	51.1 ± 18.5	54.1 ± 16.5	<0.001
BMI (kg/m^2^)	30.4 ± 7.9	28.8 ± 7.8	30.5 ± 7.9	32.0 ± 7.8	<0.001
Sex (%)					<0.001
Male	3,300 (42.2%)	1,179 (45.3%)	1,246 (47.8%)	875 (33.6%)	
Female	4,514 (57.8%)	1,426 (54.7%)	1,358 (52.2%)	1730 (66.4%)	
Race (%)					<0.001
Mexican	755 (9.7%)	194 (7.4%)	283 (10.9%)	278 (10.7%)	
Hispanics	671 (8.6%)	187 (7.2%)	244 (9.4%)	240 (9.2%)	
Non-Hispanic White	4,025 (51.5%)	1,453 (55.8%)	1,353 (52%)	1,219 (46.8%)	
Non-Hispanic Black	1731 (22.2%)	490 (18.8%)	530 (20.4%)	711 (27.3%)	
Others	632 (8.1%)	281 (10.8%)	194 (7.5%)	157 (6%)	
Education levels (%)					<0.001
≤High school	3,729 (47.7%)	1,186 (45.5%)	1,261 (48.4%)	1,282 (49.2%)	
College	2,574 (32.9%)	808 (31%)	844 (32.4%)	922 (35.4%)	
> College	1,511 (19.3%)	611 (23.5%)	499 (19.2%)	401 (15.4%)	
Marital status (%)					<0.001
Not married	6,327 (81.0%)	1997 (76.7%)	2,185 (83.9%)	2,145 (82.3%)	
Married or living with a partner	1,487 (19.0%)	608 (23.3%)	419 (16.1%)	460 (17.7%)	
Family income-poverty ratio	1.8 (1.0, 3.7)	1.9 (1.0, 3.9)	2.0 (1.1, 4.1)	1.6 (0.9, 3.2)	<0.001
Smoking status (%)					0.061
Yes	4,297 (55.0%)	1,478 (56.7%)	1,425 (54.7%)	1,394 (53.5%)	
No	3,517 (45.0%)	1,127 (43.3%)	1,179 (45.3%)	1,211 (46.5%)	

Mean ± standardized differences (SD) for continuous variables, percentages (%) for categorical variables. BMI, body mass index; TyG, Triglyceride-glucose.

### Association between TyG index and mortality

Table 2 shows the all-cause mortality of CRD patients at different quartile levels of TyG index. In model 1, without any adjustments, The HR (95% CI) from the lowest TyG group to the highest TyG group were 1.00 (reference), 1.22 (1.06–1.42), and 1.73 (1.51–1.99), respectively (P < 0.001) (Table 2). The risk of all-cause death was significantly increased (Table 2). After further adjusting for age, sex, and race in Model 1, the HR (95%CI) from the lowest TyG group to the highest TyG group were 1.00 (reference), 1.22 (1.05–1.41), and 1.74 (1.52–2.00) (P < 0.001) (Table 2). With the gradual increase of TyG index, the risk of all-cause death also gradually increased (Table 2). Following further modifications for marital status, educational attainment, body mass index, and the ratio of family income to poverty, Model 3’s outcomes resembled those of Model 2. The HR (95% confidence intervals) between the lowest TyG group and the highest TyG group were 1.00 (reference), 1.26 (1.07–1.47), and 1.63 (1.4–1.89) (P < 0.001).

**TABLE 2:** Multivariate Cox regression analysis of TyG index with all-cause mortality.

TyG index	Model 1 HR (95% CI) P-value	Model 2 HR (95% CI) P-value	Model 3 HR (95% CI) P-value
Q1	References	References	References
Q2	1.22 (1.06–1.42) 0.006	1.22 (1.05–1.41) 0.008	1.26 (1.07–1.47) 0.004
Q3	1.73 (1.51–1.99) <0.001	1.74 (1.52–2.00) <0.001	1.63 (1.4–1.89) <0.001
P for trend	<0.001	<0.001	<0.001

TyG, triglyceride-glucose; HR, hazard ratio; CI, confidence interval.

Model 1 adjust for none.

Model 2 adjust for age, sex, and race.

Model 3 adjust for age, sex, race, marital status, education level, body mass index, family income-poverty ratio, Smoking status.

**FIGURE 2:**
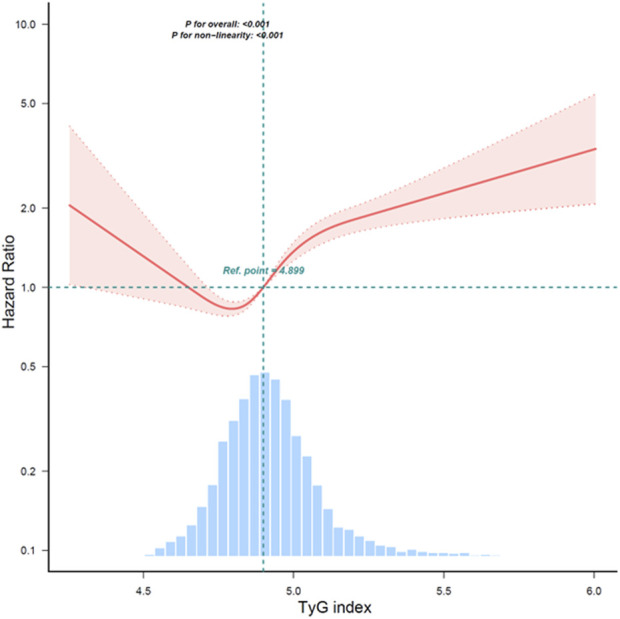
Restricted cubic spline analysis of the association between TyG and all-cause mortality.

**FIGURE 3:**
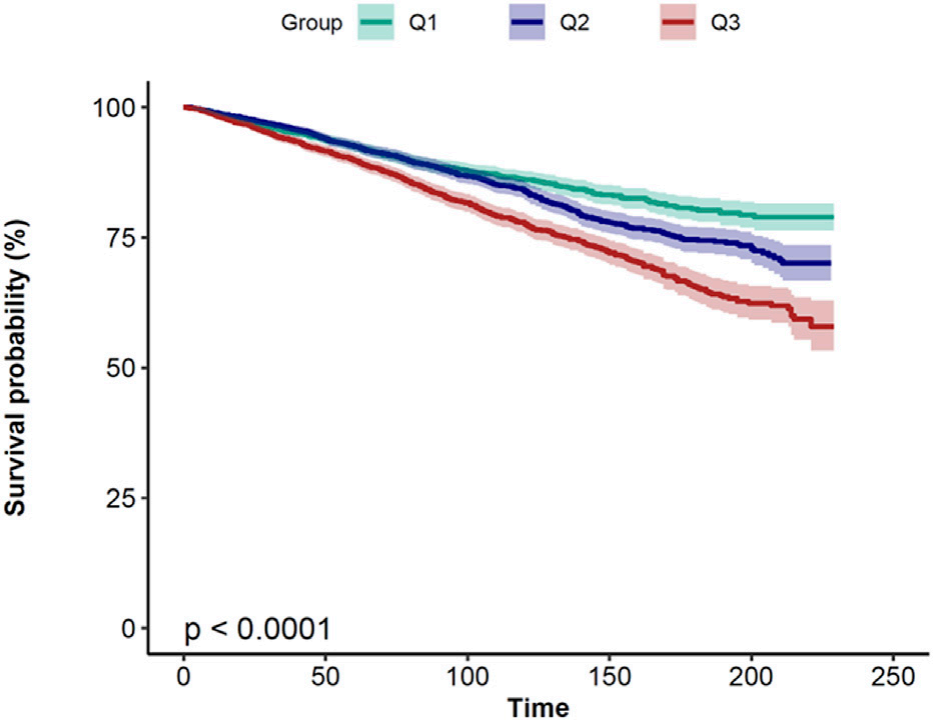
Kaplan–Meier survival analysis plot for all-cause mortality with TyG categories.

A study found that the continuous TyG index variable and overall mortality have a non-linear link after controlling for age, gender, race, marital status, education level, household income poverty rate, and other variables. A significant correlation (*P* < 0.001) was found in the examination of smoothed curve fitting plots between higher TyG index values and greater all-cause mortality in CRDs persons (Figure 2). Subsequently, Kaplan-Meier survival analysis was conducted to assess the association between TyG index and all-cause mortality. The analysis revealed that patients with the lowest TyG group exhibited the lowest lifetime risk of all-cause death (Figure 3). Other TyG-derived indices, such as TyG-BMI, which incorporates BMI to enhance the predictive power for insulin resistance. TyG-BMI was also significantly associated with the all-cause mortality in CRDs (Supplemantary Table S1).

## Discussion

Our study, involving 7,819 individuals with CRDs, demonstrates that the TyG index levels significantly and independently impact patient survival outcomes. This finding underscores the potential role of IR in chronic respiratory diseases CRDs and highlights the need for further investigation into the mechanisms underlying this relationship.

TyG index has emerged as a valuable biomarker for respiratory diseases, providing insights into the relationship between metabolic disturbances and lung pathology. This association is supported by Zaigham et al., who demonstrated that elevated TyG levels independently predict the occurrence of COPD events, reflecting its potential role as a risk marker for future respiratory complications (Zaigham et al., 2022a). Kim et al. further corroborated this relationship by presenting evidence that the risk of COPD increased in accordance with rising TyG index levels, suggesting a significant role for metabolic factors in the development of respiratory diseases (Kim et al., 2024). These findings collectively affirm the potential utility of the TyG index as a diagnostic and prognostic tool in respiratory medicine.

The impact of insulin resistance on lung function and airway inflammation severity is also not well understood. Previous research has shown a significant correlation between reduced lung function and impaired glucose regulation, as evidenced by data from the American National Health and Nutrition Examination Survey (NHANES-III) and the American Atherosclerosis Risk in Communities (ARIC) cohort (McKeever et al., 2005). Similar findings were reported in the Ansan-Ansung cohort, where IR was associated with a faster decline in forced expiratory volume in one second (FEV1) and forced vital capacity (FVC) (Kim et al., 2021). These results suggest that IR, as reflected by the TyG index, may contribute to the progression of chronic lung diseases.

Extensive research has explored the association between Metabolic Syndrome (MetS), TyG index, and pulmonary diseases, with several studies linking MetS to significant declines in lung function (Lin et al., 2006; Fimognari et al., 2007; Nakajima et al., 2008; Leone et al., 2009). Notably, Nakajima et al. demonstrated a strong association between C-reactive protein (CRP) levels and FVC in individuals with MetS (Nakajima et al., 2008). Additionally, the TyG index has emerged as a new risk marker for future chronic obstructive pulmonary disease (COPD) events in women (Zaigham et al., 2022b). Wu, et al. also found that TyG was a risk factor for impaired Lung Health (Wu et al., 2021). However, the exact biological processes underlying the relationship between death and TyG index in patients with chronic lung disease remain unclear. There are various reasons why reduced lung function is linked to MetS. MetS involves a group of cardiovascular risk factors, such as IR, dyslipidemia, glucose intolerance, and hypertension. These factors may arise from visceral obesity as a common underlying cause (Reaven, 2005). Obesity has been associated with respiratory system impairment for a long time, which can lead to restricted airflow resulting in decreased airway diameter, increased oxygen consumption during respiration, reduced compliance of the respiratory system, and heightened airway hyperresponsiveness (AHR) (Peters et al., 2018). In individuals who are obese and have low lung volume, the pulmonary retraction force on the airway is diminished leading to narrowed airway diameter and elevated AHR levels that could negatively impact lung function. The relationship between obstructive pulmonary function and MetS can be explained by mechanisms related to obesity like systemic inflammation and adipokines (Poulain et al., 2006). IR may be related to the underlying primary pathway. One of the main characteristics of the metabolic syndrome is IR, which is characterized as reduced insulin sensitivity in peripheral organs (Hunter and Garvey, 1998). TyG elevation is indicative of hyperglycemia and dyslipidemia, which alter lung structure and function. The presence of free fatty acids in the blood and hypertriglyceridemia cause macrophages and other immune cells to become inflamed (Papaioannou et al., 2017). It has also been demonstrated that the innate immune system’s lipotoxicity is triggered by LDL signaling and intracellular cholesterol buildup (Fessler, 2017; Tall and Yvan-Charvet, 2015). Reduced host immunity and increased virulence of infecting microorganisms are observed in individuals with diabetes and hyperglycemia, leading to an elevated risk and severity of lung infections (Klekotka et al., 2015). Furthermore, it has been demonstrated that insulin directly causes airway hyperresponsiveness and fibroblast collagen deposition (Park et al., 2019). All of these pathophysiological changes may lead to a poor prognosis, and they can also influence the onset and progression of chronic lung disease. By altering parasympathetic transmission, hypersensitivity to insulin and resistance to its effects—which are frequently linked to aberrant lipid levels and diabetes—have the capacity to cause greater reactivity in the bronchial airways. Furthermore, they might promote the growth of fibrosis beneath the lining of the airway (Lee et al., 2014).

Elevated TyG levels have been consistently associated with increased susceptibility to inflammatory diseases. Hypertriglyceridemia and hyperglycemia, which are key components of the TyG index, activate macrophages and promote the release of pro-inflammatory cytokines such as interleukin-6 (IL-6) and tumor necrosis factor-alpha (TNF-α), driving chronic low-grade systemic inflammation (Chen et al., 2025). This inflammatory milieu may exacerbate airway inflammation in CRDs, potentially accelerating lung tissue remodeling and functional decline. Notably, studies have demonstrated that a high TyG index correlates with elevated C-reactive protein (CRP) levels in patients with chronic obstructive pulmonary disease (COPD), suggesting a shared inflammatory pathway (Zaigham et al., 2022a). In CRDs, persistent inflammation could amplify bronchial hyperresponsiveness and mucus hypersecretion, directly contributing to mortality risk. Furthermore, metabolic disturbances reflected by the TyG index, including dyslipidemia and impaired glucose homeostasis, are linked to increased oxidative stress. Excess free fatty acids and hyperglycemia drive mitochondrial dysfunction and overproduction of reactive oxygen species (ROS), overwhelming endogenous antioxidant systems such as superoxide dismutase and glutathione (Yang et al., 2024). Oxidative damage to pulmonary epithelium and endothelial cells may impair barrier function, promote fibrosis, and reduce lung compliance—critical factors in the progression of chronic bronchitis and emphysema. This aligns with findings by Wu et al., who reported that TyG index elevation correlates with biomarkers of oxidative stress in individuals with impaired lung health (Putcha et al., 2022). Immune dysregulation represents another critical mechanism. Insulin resistance and hyperglycemia impair neutrophil phagocytic activity and alter macrophage polarization toward a pro-inflammatory phenotype, compromising host defense against respiratory pathogens. In CRD patients, this immune dysfunction may increase the frequency and severity of infections, a major driver of mortality. For instance, hyperglycemia has been associated with higher rates of bacterial pneumonia and poorer outcomes (Rosenquist et al., 2013).

Our findings have important clinical implications. The TyG index is a simple and easily calculated biomarker derived from routine clinical parameters, making it a practical tool for identifying high-risk patients with CRDs. By monitoring TyG levels, clinicians can stratify patients based on their risk of adverse outcomes and implement targeted interventions such as lifestyle modifications, glucose management, or lipid-lowering therapies. Early identification of high-risk individuals may improve prognosis and reduce mortality in this population. Additionally, the TyG index could serve as a cost-effective marker for longitudinal monitoring of disease progression and treatment response in resource-limited settings.

### Limitations and strengths

Our study has several strengths, including its large sample size, nationally representative data from the NHANES survey, and adjustment for multiple potential confounders. However, it also has limitations. As a retrospective analysis of an observational study, our findings cannot establish causality. Additionally, the cross-sectional nature of NHANES limits our ability to draw conclusions about the temporal relationship between TyG index and outcomes. Finally, the generalizability of our findings to populations outside the United States should be approached with caution.

## Conclusion

Our findings suggest that the TyG index could serve as a clinically useful biomarker for predicting survival in patients with CRDs. Given its simplicity and reliance on readily available clinical parameters, the TyG index may be a practical tool for identifying high-risk individuals and guiding targeted interventions.

## Data Availability

Publicly available datasets were analyzed in this study. This data can be found at: https://www.cdc.gov/nchs/nhanes/index.html.

## References

[B1] Chen S. Kuhn M. Prettner K. Yu F. Yang T. Bärnighausen T. (2023). The global economic burden of chronic obstructive pulmonary disease for 204 countries and territories in 2020-50: a health-augmented macroeconomic modelling study. Lancet Glob. Health 11 (8), e1183–e1193. 10.1016/S2214-109X(23)00217-6 37474226 PMC10369014

[B2] Chen X. Du X. Lu F. Zhang J. Xu C. Liang M. (2025). The association between the triglyceride–glucose index, its combination with the body roundness index, and chronic kidney disease in patients with type 2 diabetes in eastern China: a preliminary study. Nutrients 17, 492. 10.3390/nu17030492 39940350 PMC11820941

[B3] Ehrlich S. F. Quesenberry C. P. Van Den Eeden S. K. Shan J. Ferrara A. (2010). Patients diagnosed with diabetes are at increased risk for asthma, chronic obstructive pulmonary disease, pulmonary fibrosis, and pneumonia but not lung cancer. Diabetes Care 33 (1), 55–60. 10.2337/dc09-0880 19808918 PMC2797986

[B4] Fessler M. B. (2017). A new frontier in immunometabolism. Cholesterol in lung health and disease. Ann. Am. Thorac. Soc. 14 (Suppl. ment_5), S399–S405. 10.1513/AnnalsATS.201702-136AW 29161079 PMC5711269

[B5] Fimognari F. L. Pasqualetti P. Moro L. Franco A. Piccirillo G. Pastorelli R. (2007). The association between metabolic syndrome and restrictive ventilatory dysfunction in older persons. J. Gerontol. A Biol. Sci. Med. Sci. 62 (7), 760–765. 10.1093/gerona/62.7.760 17634324

[B6] GBD 2019 Diseases and Injuries Collaborators (2020). Global burden of 369 diseases and injuries in 204 countries and territories, 1990-2019: a systematic analysis for the Global Burden of Disease Study 2019. Lancet 396 (10258), 1204–1222. 10.1016/S0140-6736(20)30925-9 33069326 PMC7567026

[B7] Gläser S. Krüger S. Merkel M. Bramlage P. Herth F. J. F. (2015). Chronic obstructive pulmonary disease and diabetes mellitus: a systematic review of the literature. Respiration 89 (3), 253–264. 10.1159/000369863 25677307

[B8] Global regional (2017). Global, regional, and national deaths, prevalence, disability-adjusted life years, and years lived with disability for chronic obstructive pulmonary disease and asthma, 1990-2015: a systematic analysis for the Global Burden of Disease Study 2015. Lancet Respir. Med. 5 (9), 691–706. 10.1016/S2213-2600(17)30293-X 28822787 PMC5573769

[B9] Global burden of chronic respiratory diseases and risk factors (2023). Global burden of chronic respiratory diseases and risk factors, 1990-2019: an update from the Global Burden of Disease Study 2019. EClinicalMedicine 59, 101936. 10.1016/j.eclinm.2023.101936 37229504 PMC7614570

[B10] Hunter S. J. Garvey W. T. (1998). Insulin action and insulin resistance: diseases involving defects in insulin receptors, signal transduction, and the glucose transport effector system. Am. J. Med. 105 (4), 331–345. 10.1016/s0002-9343(98)00300-3 9809695

[B11] Kim O. H. Lee K. N. Han K. Cho I. Y. Shin D. W. Lee S. W. (2024). Association between metabolic syndrome and chronic obstructive pulmonary disease development in young individuals: a nationwide cohort study. Respir. Res. 25 (1), 414. 10.1186/s12931-024-03038-z 39593103 PMC11590416

[B12] Kim S. H. Kim H. S. Min H. K. Lee S. W. (2021). Association between insulin resistance and lung function trajectory over 4 years in South Korea: community-based prospective cohort. BMC Pulm. Med. 21 (1), 110. 10.1186/s12890-021-01478-7 33794844 PMC8017677

[B13] Klekotka R. B. Mizgała E. Król W. (2015). The etiology of lower respiratory tract infections in people with diabetes. Pneumonol. Alergol. Pol. 83 (5), 401–408. 10.5603/PiAP.2015.0065 26379004

[B14] Lee H. Kim S. R. Oh Y. Cho S. H. Schleimer R. P. Lee Y. C. (2014). Targeting insulin-like growth factor-I and insulin-like growth factor-binding protein-3 signaling pathways. A novel therapeutic approach for asthma. Am. J. Respir. Cell Mol. Biol. 50 (4), 667–677. 10.1165/rcmb.2013-0397TR 24219511 PMC5455301

[B15] Leone N. Courbon D. Thomas F. Bean K. Jégo B. Leynaert B. (2009). Lung function impairment and metabolic syndrome: the critical role of abdominal obesity. Am. J. Respir. Crit. Care Med. 179 (6), 509–516. 10.1164/rccm.200807-1195OC 19136371

[B16] Lin W.-Y. Yao C.-A. Wang H.-C. Huang K.-C. (2006). Impaired lung function is associated with obesity and metabolic syndrome in adults. Obes. (Silver Spring) 14 (9), 1654–1661. 10.1038/oby.2006.190 17030977

[B17] McKeever T. M. Weston P. J. Hubbard R. Fogarty A. (2005). Lung function and glucose metabolism: an analysis of data from the third national health and nutrition examination survey. Am. J. Epidemiol. 161 (6), 546–556. 10.1093/aje/kwi076 15746471

[B18] Nakajima K. Kubouchi Y. Muneyuki T. Ebata M. Eguchi S. Munakata H. (2008). A possible association between suspected restrictive pattern as assessed by ordinary pulmonary function test and the metabolic syndrome. Chest 134 (4), 712–718. 10.1378/chest.07-3003 18625672

[B19] Papaioannou O. Karampitsakos T. Barbayianni I. Chrysikos S. Xylourgidis N. Tzilas V. (2017). Metabolic disorders in chronic lung diseases. Front. Med. (Lausanne) 4, 246. 10.3389/fmed.2017.00246 29404325 PMC5778140

[B20] Park Y. H. Oh E. Y. Han H. Yang M. Park H. J. Park K. H. (2019). Insulin resistance mediates high-fat diet-induced pulmonary fibrosis and airway hyperresponsiveness through the TGF-β1 pathway. Exp. Mol. Med. 51 (5), 1–12. 10.1038/s12276-019-0258-7 PMC6536500 31133649

[B21] Peters U. Dixon A. E. Forno E. (2018). Obesity and asthma. J. Allergy Clin. Immunol. 141 (4), 1169–1179. 10.1016/j.jaci.2018.02.004 29627041 PMC5973542

[B22] Poulain M. Doucet M. Major G. C. Drapeau V. Sériès F. Boulet L.-P. (2006). The effect of obesity on chronic respiratory diseases: pathophysiology and therapeutic strategies. CMAJ 174 (9), 1293–1299. 10.1503/cmaj.051299 16636330 PMC1435949

[B23] Prevalence and attributable health burden of chronic respiratory diseases (2020). Prevalence and attributable health burden of chronic respiratory diseases, 1990-2017: a systematic analysis for the Global Burden of Disease Study 2017. Lancet Respir. Med. 8 (6), 585–596. 10.1016/S2213-2600(20)30105-3 32526187 PMC7284317

[B24] Putcha N. Anzueto A. Calverley P. M. A. Celli B. R. Tashkin D. P. Metzdorf N. (2022). Mortality and exacerbation risk by body mass index in patients with COPD in TIOSPIR and UPLIFT. Ann. Am. Thorac. Soc. 19 (2), 204–213. 10.1513/AnnalsATS.202006-722OC 34406915 PMC8867355

[B25] Reaven G. M. (2005). The metabolic syndrome: requiescat in pace. Clin. Chem. 51 (6), 931–938. 10.1373/clinchem.2005.048611 15746300

[B26] Rosenquist K. J. Pedley A. Massaro J. M. Therkelsen K. E. Murabito J. M. Hoffmann U. (2013). Visceral and subcutaneous fat quality and cardiometabolic risk. Jacc Cardiovasc. Imaging 6 (7), 762–771. 10.1016/j.jcmg.2012.11.021 23664720 PMC3745280

[B27] Simental-Mendía L. E. Guerrero-Romero F. (2020). The correct formula for the triglycerides and glucose index. Eur. J. Pediatr. 179 (7), 1171. 10.1007/s00431-020-03644-1 32415336

[B28] Tall A. R. Yvan-Charvet L. (2015). Cholesterol, inflammation and innate immunity. Nat. Rev. Immunol. 15 (2), 104–116. 10.1038/nri3793 25614320 PMC4669071

[B29] The L. (2018). GBD 2017: a fragile world. Lancet 392 (10159), 1683. 10.1016/S0140-6736(18)32858-7 30415747

[B30] Wu T. D. Fawzy A. Brigham E. McCormack M. C. Rosas I. Villareal D. T. (2021). Association of triglyceride-glucose index and lung health: a population-based study. Chest 160 (3), 1026–1034. 10.1016/j.chest.2021.03.056 33839084 PMC8449007

[B31] Yang M. Shangguan Q. Xie G. Sheng G. Yang J. (2024). Oxidative stress mediates the association between triglyceride-glucose index and risk of cardiovascular and all-cause mortality in metabolic syndrome: evidence from a prospective cohort study. Front. Endocrinol. 15, 1452896. 10.3389/fendo.2024.1452896 PMC11368748 39229375

[B32] Zaigham S. Tanash H. Nilsson P. M. Muhammad I. F. (2022a). Triglyceride-glucose index is a risk marker of incident COPD events in women. Int. J. Chronic Obstr. Pulm. Dis. 17, 1393–1401. 10.2147/COPD.S360793 PMC9212790 35746923

[B33] Zaigham S. Tanash H. Nilsson P. M. Muhammad I. F. (2022b). Triglyceride-glucose index is a risk marker of incident COPD events in women. Int. J. Chron. Obstruct Pulmon Dis. 17, 1393–1401. 10.2147/COPD.S360793 35746923 PMC9212790

