# Supplementary material for: Triglyceride-glucose index as an independent predictor of mortality in patients with chronic respiratory diseases
Source: Front Pharmacol. 2025 May 12;16:1474265. doi: 10.3389/fphar.2025.1474265 (PMC12104191; doi:10.3389/fphar.2025.1474265)
Supplement: Supplementary file 1 [file Table1.docx]

Supplementary Table 1. Multivariate Cox regression analysis of TyG-BMI with all-cause mortality.

| TyG index | Model 1  HR (95% CI) P-value | Model 2 HR (95% CI) P-value | Model 3 HR (95% CI) P-value |
| --- | --- | --- | --- |
| Q1 | Reference | Reference | Reference |
| Q2 | 0.91 (0.8~1.04) 0.162 | 0.91 (0.8~1.03) 0.15 | 0.88 (0.77~1.01) 0.061 |
| Q3 | 0.77 (0.67~0.88) <0.001 | 0.83 (0.72~0.95) 0.006 | 0.76 (0.66~0.88) <0.001 |
| P for trend | <0.001 | <0.001 | <0.001 |

TyG, triglyceride-glucose; BMI, body mass index; HR, hazard ratio; CI, confidence interval.

Model 1 adjust for none.

Model 2 adjust for age, sex, and race.

Model 3 adjust for age, sex, race, marital status, education level, body mass index, family income-poverty ratio, Smoking status.
